# Rotavirus gastroenteritis: epidemiological, clinical, therapeutic aspects and economic implications

**DOI:** 10.1186/1471-2334-13-S1-P88

**Published:** 2013-12-16

**Authors:** Victoria Bîrluțiu, Rareş Mircea Bîrluțiu

**Affiliations:** 1 “Lucian Blaga” University Sibiu, Faculty of Medicine Sibiu, Romania; 2 Academic Emergency Hospital Sibiu, Romania

## Background

We present the epidemiological, clinical and therapeutic aspects of rotavirus gastroenteritis, as well as its economic implications.

## Methods

We performed a prospective, observational study of cases with rotavirus enteritis admitted from 01 January 2011 to 31 December 2012 to the Infectious Diseases Clinic, Sibiu. All patients were aged 0-16 years. We followed the seasonality, the clinical aspects and the severity of the disease, laboratory tests, the need for treatment and hospitalization costs.

## Results

114 cases were diagnosed with rotavirus gastroenteritis in 2011 and 122 cases in 2012, frequently in the colder months, sex ratio M/F 1.42/1 in 2011, 1.18/1 in 2012 and in the age group 1-3 (58.90% of cases). The clinical severity was assessed by Vesikari score: 91 cases admitted in 2011 (79.82%) and 112 cases admitted in 2012 (91.80%) presenting a medium/severe score. 15 cases presented neurological symptoms and also 15 cases had renal failure. Among the electrolyte disorders the most commonly found was hyponatremia (<130 mEq/L) in 83 cases in 2011 and 51 cases in 2012, one case from the period in study was found with severe hypernatremia (>150mEq/L). The hospitalization costs of rotavirus gastroenteritis cases ranged from 58.60€ to 5345 RON (1243.02€).

## Conclusion

Gastroenteritis with rotavirus remains an important public health issue. It is necessary to widen the investigation of rotavirus gastroenteritis for easier/medium clinical forms that are treated at home in order to assess objectively the scale of the phenomenon in Romania and to support the introduction of the vaccination into the national immunization program.

